# DNA Damage Response Gene-Based Subtypes Associated With Clinical Outcomes in Early-Stage Lung Adenocarcinoma

**DOI:** 10.3389/fmolb.2022.901829

**Published:** 2022-06-22

**Authors:** Yang Zhao, Bei Qing, Chunwei Xu, Jing Zhao, Yuchen Liao, Peng Cui, Guoqiang Wang, Shangli Cai, Yong Song, Liming Cao, Jianchun Duan

**Affiliations:** ^1^ Department of Thoracic Surgery, Shanghai Chest Hospital, Shanghai Jiao Tong University, Shanghai, China; ^2^ Department of Thoracic Surgery, The Second Xiangya Hospital, Central South University, Changsha, China; ^3^ Department of Respiratory Medicine, Jinling Hospital, Nanjing University School of Medicine, Nanjing, China; ^4^ Burning Rock Biotech, Guangzhou, China; ^5^ Department of Respiratory Medicine, Xiangya Hospital, Central South University, Changsha, China; ^6^ CAMS Key Laboratory of Translational Research on Lung Cancer, State Key Laboratory of Molecular Oncology, Department of Medical Oncology, National Cancer Center/National Clinical Research Center for Cancer/Cancer Hospital, Chinese Academy of Medical Sciences Peking Union Medical College, Beijing, China

**Keywords:** DNA damage response, signature, prognostic, molecular subtype, early-stage, lung adenocarcinoma

## Abstract

DNA damage response (DDR) pathways play a crucial role in lung cancer. In this retrospective analysis, we aimed to develop a prognostic model and molecular subtype based on the expression profiles of DDR-related genes in early-stage lung adenocarcinoma (LUAD). A total of 1,785 lung adenocarcinoma samples from one RNA-seq dataset of The Cancer Genome Atlas (TCGA) and six microarray datasets of Gene Expression Omnibus (GEO) were included in the analysis. In the TCGA dataset, a DNA damage response gene (DRG)–based signature consisting of 16 genes was constructed to predict the clinical outcomes of LUAD patients. Patients in the low-DRG score group had better outcomes and lower genomic instability. Then, the same 16 genes were used to develop DRG-based molecular subtypes in the TCGA dataset to stratify early-stage LUAD into two subtypes (DRG1 and DRG2) which had significant differences in clinical outcomes. The Kappa test showed good consistency between molecular subtype and DRG (K = 0.61, *p* < 0.001). The DRG subtypes were significantly associated with prognosis in the six GEO datasets (pooled estimates of hazard ratio, OS: 0.48 (0.41–0.57), *p* < 0.01; DFS: 0.50 (0.41–0.62), *p* < 0.01). Furthermore, patients in the DRG2 group benefited more from adjuvant therapy than standard-of-care, which was not observed in the DRG1 group. In summary, we constructed a DRG-based molecular subtype that had the potential to predict the prognosis of early-stage LUAD and guide the selection of adjuvant therapy for early-stage LUAD patients.

## Introduction

Lung cancer is the major cause of global cancer mortality in 2020, with an estimated 1.8 million deaths worldwide (Sung et al., 2021). Non–small cell lung cancer (NSCLC) represents 85% of all lung cancers. Based on histology, NSCLC can be further divided into lung adenocarcinoma (LUAD), lung squamous cell carcinoma (LSCC), large-cell carcinoma, etc. (Bender, 2014). The survival of patients with NSCLC is largely determined by the tumor stage at diagnosis. Only 15% of patients with late-stage disease (stages III–IV) are alive after 5 years, which makes NSCLC one of the cancers with the worst prognosis (Necchi et al., 2017). Although the 5-year survival rate increases to approximately 60% and 40% for stage I and stage II patients, respectively, around 30–55% of them experienced disease recurrence within 5 years after surgery (Howington et al., 2013; Wang et al., 2017). In recent years, the immuno-oncology (IO)–based strategies, such as immune checkpoint inhibitors (ICIs), the combination of different ICIs, or chemotherapies, have achieved evolutionized improvements in the treatment for a subset of patients with lung cancer (Listì et al., 2019; Passiglia et al., 2021). Besides the breakthrough in cancer therapy, it is also important to improve recurrence prediction and clinical management with the increase of early-stage tumors due to the progress of lung cancer screening.

The rapid development of high-throughput technologies, especially DNA microarrays and RNA-sequencing, has facilitated the exploration of several expression-based gene signatures for risk stratification in NSCLC patients. Beer et al. proposed a 50-gene signature to identify low- and high-risk stage I lung adenocarcinomas using microarray analysis (Beer et al., 2002). The Director’s Challenge Consortium validated the performance of several such prognostic models in a large multi-site cohort with 442 lung adenocarcinomas (Chen et al., 2007; Shedden et al., 2008; Sun et al., 2008). In addition, a 14-gene expression signature (RT-PCR–based) has been commercialized to stratify different risk groups for resected non-squamous NSCLC patients (Kratz et al., 2012). A 25–immune gene signature and a 31–proliferation gene signature both have shown promising clinical utility for risk stratification and individualized management in NSCLC patients (Wistuba et al., 2013; Li et al., 2017). However, none of these signatures was further analyzed in patients with and without adjuvant therapy to validate the potential clinical utility in the guidance of adjuvant therapy.

Genomic instability is one of the key hallmarks of cancer, and DNA damage response (DDR) plays a significant role in maintaining genomic integrity (Hanahan and Weinberg, 2011). The DDR system is a complex signaling network which involves eight pathways: base excision repair (BER), mismatch repair (MMR), homologous recombination repair (HRR), nonhomologous end joining (NHEJ), checkpoint factors (CPF), Fanconi anemia (FA), nucleotide-excision repair (NER), and DNA translesion synthesis (TLS) (Scarbrough et al., 2016). These pathways operate collectively to detect diverse types of DNA lesions and activate signaling mechanisms to boost the repair machine (Jackson and Bartek, 2009). Previous studies have demonstrated that the DDR pathways play significant roles in cancer progression and the response to cancer therapies. Several prognostic models, based on DDR genes, have been constructed for glioblastoma, ovarian cancer, and low-grade gliomas (Knijnenburg et al., 2018; Gobin et al., 2019; Sun et al., 2019; Pang et al., 2020). However, the DDR genes identified in these prognostic models vary widely between different cancers, suggesting that DDR genes may exert different molecular effects in different cellular environments. The relationships of various DDR genes with prognosis in lung adenocarcinoma are not well-established.

In this study, we aimed to identify and validate a group of DDR genes to stratify early-stage LUAD patients into different subtypes with different prognoses and guide the use of adjuvant therapy.

## Materials and Methods

### Molecular and Clinical Data

The LUAD dataset of The Cancer Genome Atlas (TCGA) and six microarray datasets of Gene Expression Omnibus (GEO) were included in the analysis. For the TCGA dataset, RNA-sequencing data (FPKM format), genetic mutations, copy number variant (CNV), and clinical features, including age, sex, tumor stage, histology subtype, adjuvant treatment, and follow-up information, were obtained from the GDC (https://portal.gdc.cancer.gov/). In addition, normalized microarray data and the corresponding clinical characteristics of patients with early-stage (stages I and II) lung adenocarcinoma from six GEO cohorts (GSE31210, GSE37745, GSE68465, GSE30219, GSE72094, and GSE13213) were obtained for further external validation in this study.

### DDR Gene–Based Signature Construction

A total of 200 DDR-related genes were curated and analyzed to identify prognosis-related markers (Scarbrough et al., 2016). These genes used in the study are listed in Supplementary Table S1. Univariable Cox regression and LASSO Cox regression analyses with minimum partial likelihood deviance were performed to select genes associated with OS. We defined the risk score using the following formula

$$riskscore=\overset{n}{\underset{k=0}{\sum }}(coef\;of\;genek*expr\;of\;genek\;),$$

where *n* is the number of markers. The nearest neighbor estimation method was applied to identify the best cutoff point of risk score to stratify patients into high- and low-risk subgroups. Kaplan–Meier (KM) analysis and receiver operating characteristic (ROC) curve were used to assess the performance of the signature.

### Association Between DDR Signature and Genome Features and Gene Expression

In order to explore the potential molecular mechanisms of the DDR-gene–based signature, the associations of the DDR signature with somatic mutation, CNV, genomic scar signature, and gene expression data were analyzed based on TCGA data.

### DDR Molecular Subtype Identification and Validation

Unsupervised clustering with the hierarchical cluster algorithm (based on Euclidean distance and Ward’s linkage) of the expression profiles of the genes in the DDR signature was performed to identify molecular subtype in early-stage LUAD. The default parameters of the hclust function were used to perform the classification. The cluster number was selected as 2. It was further validated in six GEO datasets.

### Statistical Analyses

R software v4.0.2 was used for all the bioinformatics and statistical analyses, including data preprocess, LASSO Cox regression, CNV and mutation visualization, and ROC analysis. The KM method and log-rank test were adopted to generate and evaluate the statistical significance of the survival curves between groups. The specificity and sensitivity of the signature were evaluated using the ROC curve, and the area under the curve (AUC) of distinct survival time was quantified using R-package pROC. The Kappa consistency test was used to analyze the consistency between the two group methods. The Cox proportional hazards model was applied to identify the independence of the signature. The prognostic values of single genes in signatures were accessed using the “szcox” function of the ezcox package. R-package SubgrPlots was used for subgroup analysis, which was visualized using the Forester package. A propensity score matching (PSM) analysis was performed according to a 1:1 ratio between the two subgroups (with or without adjuvant therapy) to adjust for clinicopathologic characteristics bias using the MatchIt package. Heat maps of TCGA–LUAD and GEO datasets were generated using the pheatmap package. The maftools package was used to visualize the mutation landscape in the TCGA–LUAD dataset. Two-sided *p* < 0.05 was considered to be statistically significant.

## Results

### Construction of a 16-Gene Signature

**FIGURE 1:**
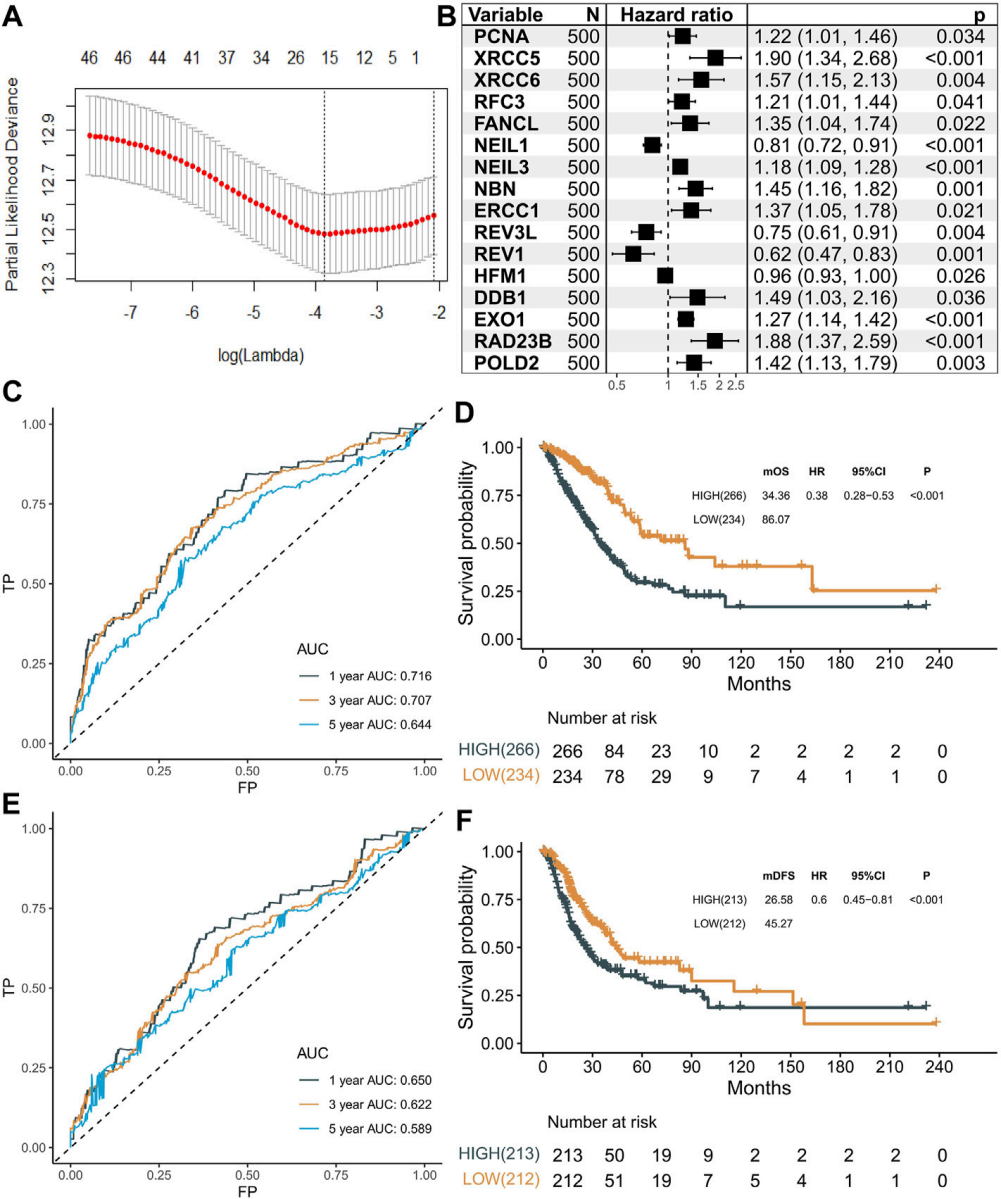
Selection of prognostic markers. **(A)** Tuning parameter (λ) selection in the LASSO model using 10-fold cross-validation via minimum criteria. **(B)** Forest plot showing the results of univariable Cox regression analyses. **(C)** Predictive value of 16 genes in the overall survival of patients in the LUAD dataset. **(D)** Kaplan–Meier curves of overall survival for high- and low-risk patient groups in the TCGA–LUAD dataset. Patients were divided into two groups with a cutoff score of 21.96. **(E)** Predictive value of 16 genes in the disease-free survival of patients in the LUAD dataset. **(F)** Kaplan–Meier curves of disease-free survival for high- and low-risk patient groups in the TCGA–LUAD dataset. Patients were divided into two groups with a cutoff score of 21.96.

A total of 1,785 primary LUAD tumors and their clinicopathological features were downloaded from TCGA and GEO databases, and the baseline characteristics are summarized in Supplementary Table S2. To identify the survival-related genes, univariable Cox regression was performed in the 200 DDR-related genes with the TCGA–LUAD dataset (n = 500), and 46 DDR genes were identified to be significantly associated with OS. Then, ten-fold cross-validation of LASSO Cox was implemented using the “glmnet” package, and 16 genes (*PCNA*, *XRCC5*, *XRCC6*, *RFC3*, *FANCL*, *NEIL1*, *NEIL3*, *NBN*, *ERCC1*, *REV3L*, *REV1*, *HFM1*, *DDB1*, *EXO1*, *RAD23B*, and *POLD2*) were identified to be the most informative and were used to construct a risk score (Figure 1A). In brief, *NEIL1*, *HFM1*, *REV3L*, and *REV1* genes were protective factors (all HRs < 1, *p* < 0.05), while the others genes were risk factors for the prognosis in patients with LUAD (all HRs > 1, *p* < 0.05) (Figure 1B). Then, we established a DNA damage response gene (DRG)–based signature for each patient based on the following formula: DRG = (−0.0618*PCNA)+(0.2175*XRCC5)+(0.1094*XRCC6)+(−0.0929*RFC3)+(0.1894*FANCL)+(−0.0008*NEIL1)+(0.0095*NEIL3)+(0.1651*NBN)+(0.1594*ERCC1)+(−0.0817*REV3L)+(−0.0640*REV1)+(−0.0119*HFM1)+(0.1600*DDB1)+(0.1070*EXO1)+(0.2896*RAD23B)+(0.0548*POLD2). The best cutoff of 21.96 was used to stratify the patients into high- or low-risk groups. The AUCs for 1-, 3-, and 5-year overall survival (OS) rate predictions for the DRG of the TCGA–LUAD dataset were 0.716, 0.707, and 0.644, respectively (Figure 1C). The KM curves revealed significantly higher OS with lower DRG (HR = 0.38, 95% CI: 0.28–0.53, *p* < 0.001, Figure 1D). Similar results for disease-free survival (DFS) were obtained. The AUCs for 1-, 3-, and 5-year were 0.650, 0.622, and 0.589, respectively, (Figure 1E) and the association between the DRG and DFS was significant (log-rank *p* < 0.001; HR = 0.60, 95% CI: 0.45–0.81, Figure 1F). Next, we tested the independent prognostic prediction value of the DRG. After adjusting for clinical features, including age, sex, tumor stage, and smoking, as well as the driver gene mutation (EGFR, KRAS, ALK, ROS1, BRAF, and TP53), the DRG served as an independent prognostic biomarker for predicting outcomes (OS, HR: 0.42 (0.30–0.58); DFS, HR: 0.43 (0.31–0.53), Table 1).

**TABLE 1:** Univariable analysis and multivariable Cox regression analyses of OS and DFS in TCGA cohorts.

	Characteristics	Sample size	OS				DFS			
Cohort			Univariable Cox		Multivariable Cox		Univariable Cox		Multivariable Cox	
			HR (95% CI)	*P*-value	HR (95% CI)	*P*-value	HR (95% CI)	*P*-value	HR (95% CI)	*P*-value
TCGA										
—	Age (≤60 vs. >60)	490	0.86 (0.62–1.18)	0.3400	—	—	1.03 (0.77–1.38)	0.8590	0.69 (0.49–0.96)	0.0295
—	Sex (Male vs. Female)	500	1.05 (0.78–1.40)	0.7530	—	—	0.59 (0.41–0.83)	0.0031	—	—
—	Stage (I_II vs. III_IV)	492	0.39 (0.28–0.53)	<0.001	0.43 (0.31–0.58)	<0.001	1.00 (0.65–1.53)	0.9970	0.66 (0.46–0.95)	0.0271
—	Smoking (Never vs. Ever)	486	1.14 (0.75–1.72)	0.5450	—	—	2.19 (1.18–4.06)	0.0130	—	—
—	EGFR (MUT vs. WT)	383	2.38 (1.31–4.34)	0.0005	3.08 (1.66–5.71)	<0.001	0.84 (0.56–1.27)	0.4130	2.55 (1.33–4.92)	0.0050
—	KRAS (MUT vs. WT)	383	0.87 (0.55–1.36)	0.5310	—	—	0.99 (0.41–2.42)	0.9850	—	—
—	ALK (MUT vs. WT)	383	0.60 (0.19–1.89)	0.3820	—	—	1.04 (0.73–1.47)	0.8420	—	—
—	TP53 (MUT vs. WT)	383	1.23 (0.84–1.80)	0.2790	—	—	0.90 (0.49–1.68)	0.7940	—	—
—	BRAF (MUT vs. WT)	383	0.68 (0.31–1.46)	0.3200	—	—	0.60 (0.45–0.81)	0.0007	—	—
—	DRG (low vs. high)	500	0.38 (0.28–0.53)	<0.001	0.42 (0.30–0.58)	<0.001	0.72 (0.52–1.00)	0.0479	0.58 (0.43–0.79)	0.0005

### Association Between the 16 Genes and Clinicopathological Factors

**FIGURE 2:**
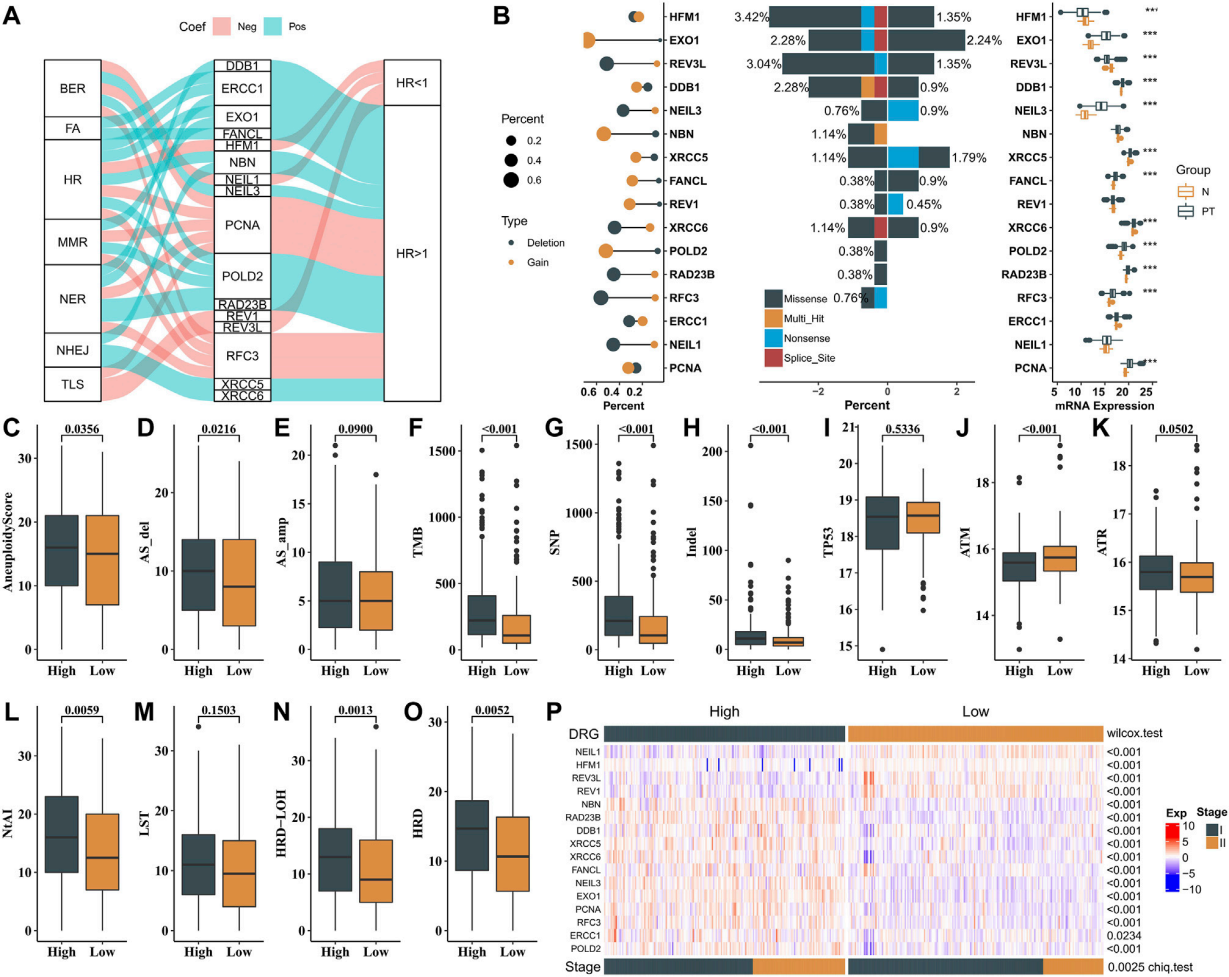
Mechanism of validation in mutation, CNV, mRNA expression, and genome instability. **(A)** Sankey plot showing the correlations among 16 genes, DDR pathways, and prognostic value. **(B)** Left panel is the CNV variation frequency of 16 genes, and the deletion frequency is shown by gray dots; the middle panel is the mutation frequency of 16 genes between high- and low-DDR score samples. The right panel is the expression of 16 genes between normal tissues and tumor tissues. Tumor, gray; normal, yellow. **(C**–**O)** Comparison of the genome instability (NtAI, LST, LOH, HRD, Aneuploidy Score (AS), AS_del, AS_amp, TMB, SNP, and indel) and expression pattern of TP53, ATM, and ATR between high-risk and low-risk patients in the TCGA dataset. High, gray; low, yellow. **(P)** Expression profiles of 16 genes between high- and low-risk groups in the stages I and II TCGA–LAUD dataset. *p*-value of continuous variables was tested using Wilcoxon rank-sum test. Pearson’s chi-square test was used to test the categorical variables.

To further study the underlying mechanism of the DRG, we explored the molecular function and the association with prognosis of genes in the DRG. Most of them had positive coefficients in this regression equation with HR > 1, indicating poor prognostic genes, while genes (*REV3L*, *REV1*, *NEIL1*, and *HFM1*) had negative coefficients with HR < 1 (Figure 2A). To depict the genomic and expression alterations of the 16 DDR genes, we further described the prevalence of somatic mutations, CNV, and mRNA expression of the 16 genes in LUAD patients (Figure 2B). Of the 486 LUAD patients, 63 (13.0%) patients harbored at least one mutation of the pattern genes. Among them, *HFM1* had the highest mutation frequency (4%) followed by *EOX1* and *REV3L*, while there were no mutations in *ERCC1*, *NEIL1*, and *PCNA*. Meanwhile, CNV analysis showed that *EXO1*, *NBN*, and *POLD2* had a widespread frequency of CNV gain. Furthermore, the mRNA expressions for these genes were significantly higher in patients with CNV gain, suggesting CNV alteration may be a vital contributor to the altered mRNA expression of these genes. Moreover, protein expression levels of 13 genes were obtained from The Human Protein Atlas (THPA). Representative IHC images revealed that these proteins had upregulated expression in lung adenocarcinoma tissues and downregulated expression in normal lung tissues (Supplementary Figure S1 and Supplementary Table S3).

To identify the biological significance of the genes in the DRG signature, GO analysis was conducted, and the results revealed that these genes were enriched in DNA-dependent DNA replication, nucleotide-excision repair, DNA recombination, and DNA geometric change. Furthermore, the outcomes of KEGG pathway analysis illustrated that these genes were mainly enriched in the Fanconi anemia pathway, base excision repair, and homologous recombination (Supplementary Figure S2).

Next, we investigated the associations between the two groups and various genomic features. The high-risk group was associated with higher aneuploidy score (AS), tumor mutational burden (TMB), SNP, and indel burden than the low-risk group (Figure 2C–H). Higher mRNA expression of ATM was observed in the low-risk group than in the high-risk group, while not for TP53 and ATR (Figure 2I–K). We also observed that samples in the high-risk group exhibited higher genomic instability—telomeric allelic imbalance (TAI), large-scale state transitions (LST), loss of heterozygosity (LOH), and an incorporated homologous recombination deficiency (HRD) score (Figure 2L-O). These results showed the heterogeneity in genomic scar and DDR checkpoint gene expression between the two groups.

### Molecular Subtype Identification

As shown in Figure 2P, two expression patterns of the 16 genes were identified from the expression heat map of these signature genes in patients with stages I and II LUAD from the TCGA cohort. Patients in the low-risk group had better clinical outcomes (OS and DFS) and showed significantly higher expressions of *REV1*, *REV3L*, *HFM1*, and *NEIL1*, while the other genes had significantly lower expressions in this group. Meanwhile, the low-risk group had a higher percentage of patients with stage I than in the high-risk group (Chi test, *p* = 0.0025). The abovementioned results demonstrated that the DRG-related genes could be used to classify the early-stage LUAD patients.

**FIGURE 3:**
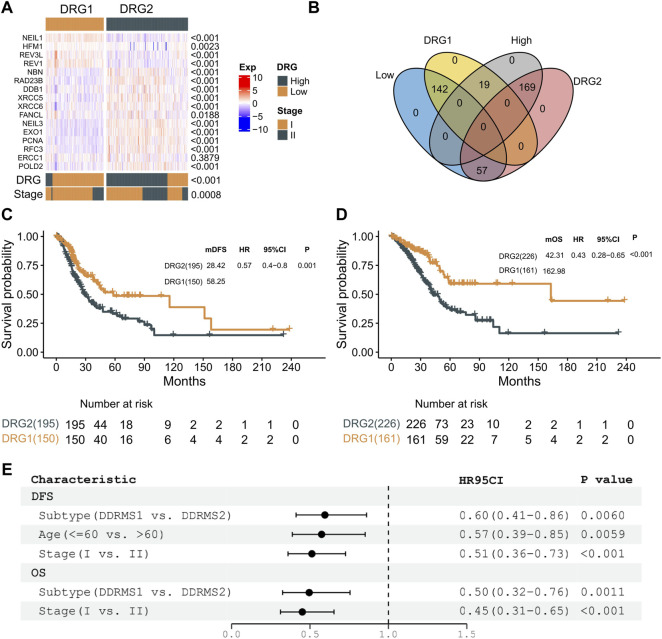
Molecular subtype identification. **(A)** Expression profiles of 16 genes between DRG1 and DRG2 in the stages I and II TCGA–LAUD dataset. *p*-value of continuous variables was tested using the Wilcoxon rank-sum test. The consistency of DRG and molecular subtype was tested using the Kappa consistency test. Pearson’s chi-square test was used to test the categorical variables. **(B)** Venn plot presenting the intersection of patient share by molecular subtype and DRG. **(C)** Kaplan–Meier curves showing DFS between DRG1 (yellow) and DRG2 (gray) in patients with early-stage LUAD. **(D)** Kaplan–Meier curves showing OS between DRG1 (yellow) and DRG2 (gray) in patients with early-stage LUAD. **(E)** Multivariable analysis of DFS and OS with a Cox proportional hazards model in early-stage lung carcinoma.

Unsupervised hierarchical clustering (based on Euclidean distance and Ward’s linkage) of the expression profiles of DDR genes was used to identify molecular subtype instead of the formula derived from the TCGA cohort. The expression profile of the 16 genes was used to develop a DRG-related molecular subtype to stratify early-stage LUAD into two subtypes (DRG1 and DRG2) with statistically significant differences in clinical outcomes. A clustering heat map was generated to illustrate that the expressions of DRG-related genes were significantly different between the two subtypes (Figure 3A). The Kappa consistency test revealed the consistency of the two methods (DRG and molecular subtype, K = 0.61, *p* < 0.001, Figure 3A). As shown in Figure 3B, 71.4% (142/199) of the low-risk DRG patients were grouped into DRG1 subtype, and 89.9% (169/188) of the high-risk DRG patients were grouped into DRG2 subtype. Similar results were also documented in the Kaplan–Meier analysis (DFS, log-rank *p* = 0.001; HR = 0.57, 95% CI: 0.40–0.80; OS, log-rank *p* < 0.001; HR = 0.43, 95% CI: 0.28–0.65, Figure 3C,D). After adjusting for clinical factors, the molecular subtype remained an independent prognostic molecular classifier for DFS and OS (DFS, HR = 0.60, 95% CI: 0.41–0.86, *p* = 0.006; OS, HR = 0.50, 95% CI: 0.32–0.76, *p* = 0.011, Figure 3E). These results indicated that DRG-related genes could stratify early-stage LUAD into two molecular subtypes with distinct prognosis.

### Validation in GEO Datasets and Meta-analysis

**FIGURE 4:**
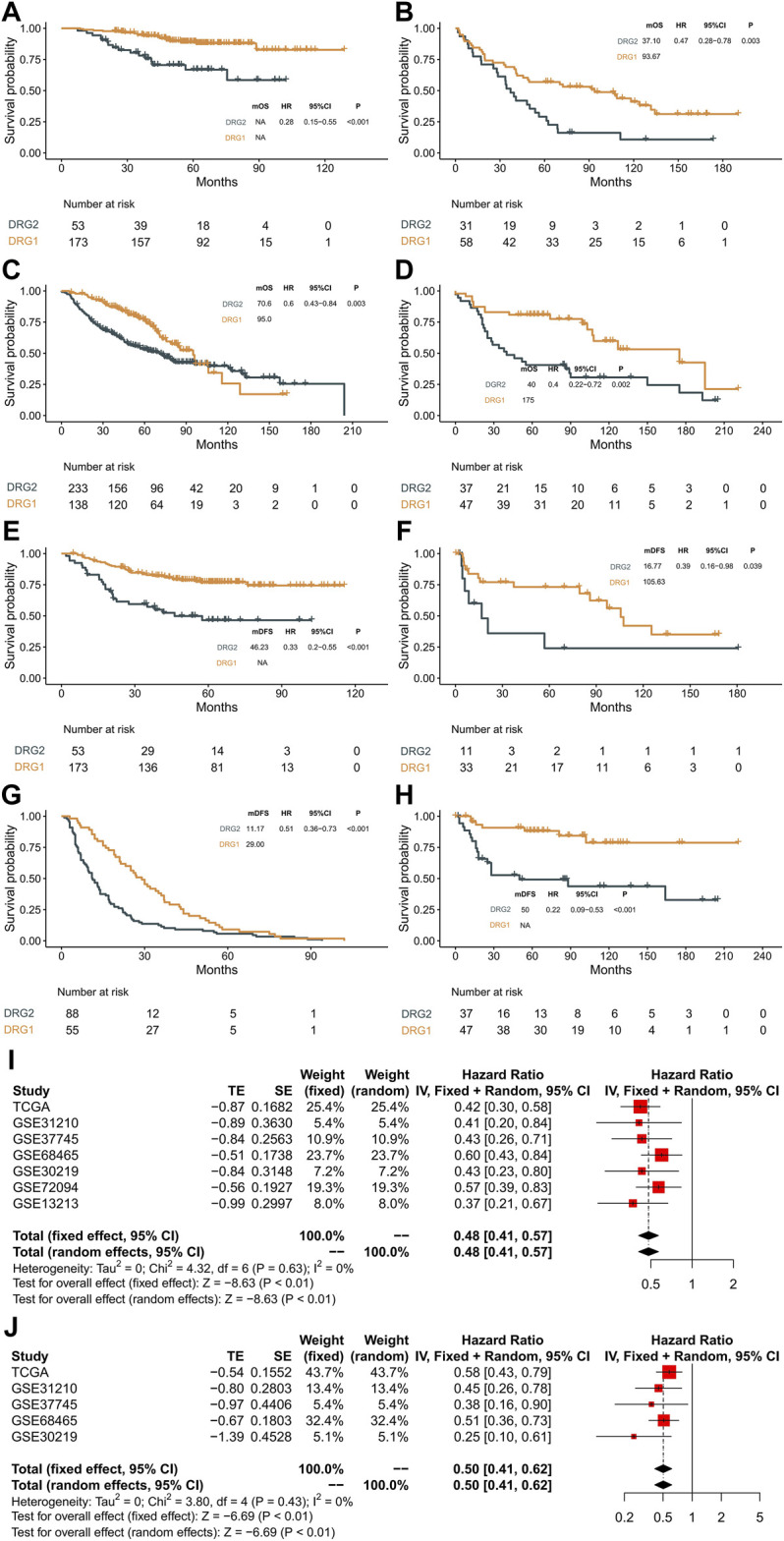
Validation in the GEO datasets and meta-analysis. **(A**–**D)** Kaplan–Meier curves showing overall survival between DRG1 (yellow) and DRG2 (gray) in GSE31210, GSE37745, GSE68465, and GSE30219. **(E**–**H)** Kaplan–Meier curves showing disease-free survival between DRG1 (yellow) and DRG2 (gray) in GSE31210, GSE37745, GSE68465, and GSE30219. **(I)** Pooled estimates of overall survival. **(J)** Pooled estimates of disease-free survival.

In order to validate the molecular subtype and prognostic prediction of the DRG-related genes, a total of 1,285 stage III LUAD patient RNA expression microarray data were collected. The expression patterns of these genes and the survival status of patients in each GEO dataset are shown in Figure 4 and Supplementary Figure S3. The patients in the DRG1 subtype had a longer OS and DFS than those in the DRG2 subtype (GSE31210: OS, log-rank *p* < 0.001, HR = 0.28, 95% CI: 0.15–0.55; DFS, log-rank *p* < 0.001, HR = 0.33, 95% CI: 0.20–0.55. GSE37745: OS, log-rank *p* = 0.003, HR = 0.47, 95% CI: 0.28–0.78; DFS, log-rank *p* = 0.039, HR = 0.39, 95% CI: 0.16–0.98. GSE68465: OS, log-rank *p* = 0.003, HR = 0.60, 95% CI: 0.43–0.84; DFS, log-rank *p* < 0.001, HR = 0.50, 95% CI: 0.37–0.68. GSE30219: OS, log-rank *p* = 0.002, HR = 0.40, 95% CI: 0.22–0.72; DFS, log-rank *p* < 0.001, HR = 0.22, 95% CI: 0.09–0.53. GSE72094: OS, log-rank *p* = 0.002, HR = 0.49, 95% CI: 0.32–0.77. GSE13213: OS, log-rank *p* < 0.001, HR = 0.22, 95% CI: 0.01–0.49).

A meta-analysis was performed with a fixed-effects model, and the results indicated that compared with the DRG2 subtype, patients with the DRG1 subtype exhibited higher OS (HR = 0.48, 95% CI: 0.41–0.57, *p* < 0.01, Figure 4I) and DFS (HR = 0.5, 95% CI: 0.41–0.62, *p* < 0.01, Figure 4J) in the overall dataset. Heterogeneities were not significant in all pooled analyses (OS, *p* = 0.63; DFS, *p* = 0.43).

Then, we tested whether the molecular subtype could serve as an independent prognostic factor for early-stage lung adenocarcinoma. In multivariable analysis, the associations of DDR subtypes and prognosis were still significant (Tables 2, 3), which confirmed that the selected DDR genes could stratify patients with different prognoses.

**TABLE 2:** Univariable analysis and multivariable Cox regression analyses of OS in six validation cohorts.

Cohort	Characteristics	Sample size	Univariable Cox		Multivariable Cox	
			HR (95% CI)	*P*-value	HR (95% CI)	*P*-value
GSE31210						
—	Age (≤60 vs. >60)	226	0.79 (0.4–1.54)	0.4860	—	—
—	Sex (Male vs. Female)	226	1.52 (0.78–2.96)	0.2190	—	—
—	Stage (II vs. I)	226	4.23 (2.17–8.24)	<0.001	3.23 (1.59–6.55)	0.0012
—	Smoking (Never vs. Ever)	226	0.61 (0.31–1.19)	0.1500	—	—
—	Adjuvant (Y vs. N)	226	2.04 (0.79–5.27)	0.1420	—	—
—	Subtype (DRG1 vs. DRG2)	226	0.28 (0.15–0.55)	0.0002	0.41 (0.2–0.83)	0.0139
GSE37745						
—	Age (≤60 vs. >60)	106	0.82 (0.52–1.3)	0.3980	—	—
—	Sex (Male vs. Female)	106	1.26 (0.8–1.97)	0.3160	—	—
—	Stage (III_IV vs. I_II)	106	1.79 (1.01–3.17)	0.0449	2.35 (1.28–4.3)	0.0056
—	Subtype (DRG1 vs. DRG2)	106	0.51 (0.32–0.82)	0.0053	0.43 (0.26–0.71)	0.0009
GSE68465						
—	Age (≤60 vs. >60)	371	0.53 (0.37–0.74)	0.0002	0.49 (0.34–0.7)	0.0001
—	Sex (Male vs. Female)	371	1.43 (1.06–1.93)	0.0198	1.52 (1.11–2.09)	0.0097
—	Smoking (Never vs. Ever)	297	1.13 (0.67–1.91)	0.6360	—	—
—	Subtype (DRG1 vs DRG2)	371	0.6 (0.43–0.84)	0.0030	0.6 (0.43–0.85)	0.0036
GSE30219						
—	Age (≤60 vs. >60)	84	0.66 (0.36–1.21)	0.1780	—	—
—	Sex (Male vs Female)	84	1.05 (0.51–2.19)	0.8870	—	—
—	Stage (II vs I)	84	2.04 (1.04–3.97)	0.0370	1.68 (0.85–3.34)	0.1370
—	Subtype (DRG1 vs DRG2)	84	0.4 (0.22–0.72)	0.0027	0.43 (0.23–0.79)	0.0068
GSE72094						
—	Age (≤60 vs >60)	398	0.72 (0.42–1.23)	0.2300	—	—
—	Sex (Male vs Female)	398	1.55 (1.07–2.25)	0.0198	1.78 (1.22–2.6)	0.0028
—	Stage (III_IV vs I_II)	393	2.61 (1.74–3.91)	<0.001	—	—
—	Smoking (Never vs Ever)	331	0.73 (0.32–1.68)	0.4590	2.81 (1.86–4.24)	<0.001
—	Subtype (DRG1 vs DRG2)	398	0.56 (0.39–0.81)	0.0023	0.57 (0.39–0.83)	0.0030
GSE13213						
—	Age (≤60 vs >60)	117	0.96 (0.55–1.69)	0.8900	—	—
—	Sex (Male vs Female)	117	1.36 (0.77–2.39)	0.2860	—	—
—	Stage (II vs I)	92	1.57 (0.64–3.82)	0.3240	1.38 (0.57–3.38)	0.4780
—	Stage (III vs I)	104	3.2 (1.74–5.88)	0.0002	2.73 (1.48–5.05)	0.0014
—	Smoking (Never vs Ever)	117	1.36 (0.77–2.39)	0.2840	—	—
—	Subtype (DRG1 vs DRG2)	117	0.33 (0.18–0.6)	0.0002	0.37 (0.21–0.68)	0.0012

**TABLE 3:** Univariable analysis and multivariable Cox regression analyses of DFS in four validation cohorts.

Cohort	Characteristics	Sample size	Univariable Cox		Multivariable Cox	
			HR (95% CI)	*P*-value	HR (95% CI)	*P*-value
GSE31210						
—	Age (≤60 vs >60)	226	0.61 (0.37–1.02)	0.0575	—	—
—	Sex (Male vs Female)	226	1.27 (0.78–2.07)	0.3380	—	—
—	Stage (II vs I)	226	3.16 (1.92–5.21)	0.0000	2.4 (1.36–4.21)	0.0024
—	Smoking (Never vs Ever)	226	0.75 (0.46–1.23)	0.2520	—	—
—	Adjuvant (Y vs N)	226	2.37 (1.2–4.66)	0.0127	1.07 (0.51–2.26)	0.8540
—	Subtype (DRG1 vs DRG2)	226	0.33 (0.2–0.55)	0.0000	0.45 (0.26–0.78)	0.0044
GSE37745						
—	Age (≤60 vs >60)	53	1.23 (0.56–2.71)	0.6140	—	—
—	Sex (Male vs Female)	53	1.07 (0.48–2.38)	0.8680	—	—
—	Stage (III_IV vs I_II)	53	1.72 (0.64–4.61)	0.2800	2.06 (0.75–5.64)	0.1610
—	Subtype (DRG1 vs DRG2)	53	0.42 (0.18–0.98)	0.0441	0.38 (0.16–0.9)	0.0282
GSE68465						
—	Age (≤60 vs >60)	143	1.01 (0.7–1.44)	0.9700	—	—
—	Sex (Male vs Female)	143	1.05 (0.76–1.47)	0.7570	—	—
—	Smoking (Never vs Ever)	130	1.02 (0.63–1.66)	0.9210	—	—
—	Subtype (DRG1 vs DRG2)	143	0.51 (0.36–0.73)	0.0002	0.51 (0.36–0.73)	0.0002
GSE30219						
—	Age (≤60 vs >60)	84	0.83 (0.38–1.79)	0.6280	—	—
—	Sex (Male vs Female)	84	1.17 (0.44–3.11)	0.7480	—	—
—	Stage (II vs I)	84	3.35 (1.49–7.57)	0.0036	2.71 (1.19–6.19)	0.0180
—	Subtype (DRG1 vs DRG2)	84	0.22 (0.09–0.53)	0.0007	0.25 (0.1–0.59)	0.0018

### Subgroup Analysis

**FIGURE 5:**
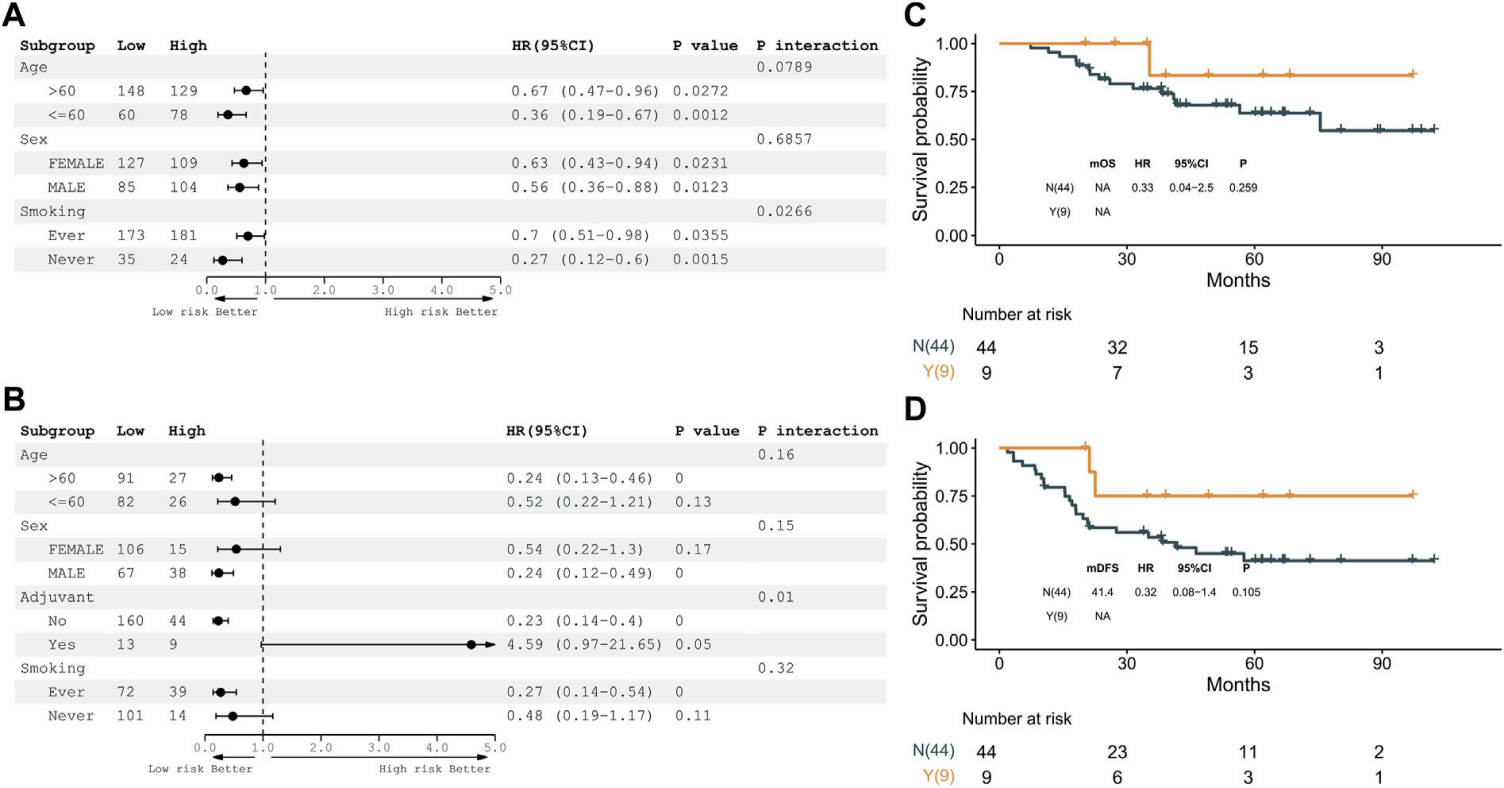
Expression pattern of 16 genes is a prognostic biomarker and predicts adjuvant therapy benefits in the GSE31210 dataset. **(A)** Subgroup analyses of overall survival to estimate the clinical prognostic value between DRG1 and DRG2 as independent clinical factors. **(B)** Subgroup analyses of disease-free survival to estimate the clinical prognostic value between DRG1 and DRG2 in independent clinical factors. **(C)** Kaplan–Meier curves of overall survival between patients treated with or without adjuvant therapy. **(D)** Kaplan–Meier curves of disease-free survival between patients treated with or without adjuvant therapy.

A stratification analysis was conducted to assess whether clinical factors had interaction effects on the DRG subtypes. Patients in TCGA and GSE31210 datasets were artificially stratified based on clinical factors, such as age (≤60/>60), sex (female/male), stage (I/II), smoking (ever/never), and adjuvant treatment (no/yes). As shown in Figure 5A,B and Supplementary Figure S4A-B, patients in the DRG1 subtype had higher OS and DFS than the DRG2 subtype irrespective of their age, sex, and smoking status. Meanwhile, a significant interaction (*p* = 0.01) between adjuvant treatment and DRG subtypes was observed in early-stage LUAD patients. Furthermore, we examined the association between adjuvant treatment and prognosis in DRG1 and DRG2 subtypes. We found that in the DRG2 subtype, patients with adjuvant treatment tended to have longer OS and DFS than patients without adjuvant treatment (OS, log-rank *p* = 0.259, HR = 0.33, 95% CI: 0.04–2.5; DFS, log-rank *p* = 0.105, HR = 0.32, 95% CI: 0.08–1.4), while in the DRG1 subtype, the results were opposite (OS, log-rank *p* = 0.001, HR = 5.3, 95% CI: 1.7–16; DFS, log-rank *p* < 0.001, HR = 6.7, 95% CI: 3.0–15). The abovementioned observation was not statistically significant because the sample size was limited (Supplementary Figure S4C,D and Figure 5C,D). After the patients were matched by propensity score, similar results were observed (Supplementary Figure S5).

## Discussion

In this study, we trained and validated 16 DDR genes with prognostic values and classification effects in early-stage lung adenocarcinoma and classified patients into two subtypes, DRG1 and DRG2. Furthermore, we found that the DRG1 patients without adjuvant therapy and the DRG2 patients with adjuvant therapy tended to have prolonged survival than other patients in the corresponding subtypes.

The DDR system comprised eight pathways with diverse biological functions to maintain genomic integrity. In this study, we discovered that the 16 identified DDR genes were mainly involved in TLS, NER, and BER pathways. *REV3L* and *REV1* were involved in TLS whose lower expressions were associated with worse prognoses. In human cells, when the expressions of TLS genes decrease, the DNA replication stress escalates the accumulative fork stalling and double-strand breaks (DSBs), resulting in genome instability and poor survival (Ghosal and Chen, 2013). These two genes are important DNA polymerase and deoxycytidyl transferase, which play significant roles in maintaining genome stability in the advent of DNA damage (Sasatani et al., 2020; Zhou et al., 2020). It has been reported that lower *REV3L* expression was also shown to be associated with lower DFS and OS (Agulló-Ortuño et al., 2020), which was consistent with our findings.

Furthermore, we discovered that genes with higher expression in the DRG2 subtype were mainly involved in NER and BER pathways. RAD23B, DDB1, and ERCC family genes (ERCC1, ERCC5, and ERCC6) are key genes in the NER pathway. Many studies have reported that they are significantly correlated with prognosis in different cancer types, such as colorectal cancer, pancreatic cancer, gastric cancer, and so on (Luo et al., 2018; Zhang et al., 2019; Li et al., 2021). *NEIL3*, *PCNA*, *RFC3*, and *POLD2* play important roles in the BER pathway which are recruited to DNA lesions and cleave and repair the damaged bases cooperatively (Robertson et al., 2009; Hurst et al., 2021; Wang et al., 2021). Zhao et al. found that *NEIL3* activated cell cycle progression, leading to poor prognosis (Zhao et al., 2021). Zhang et al. discovered that RFC3 was involved in the epithelial–mesenchymal transition in lung adenocarcinoma, resulting in worse survival (Gong et al., 2019). In tumorigenesis, increased DNA replication stress results in the increased generation of reactive oxygen species (ROS), leading to DNA damage (Jackson and Bartek, 2009). Accumulating evidence supports that NER and BER pathways are involved in the repair of oxidative DNA lesions. Therefore, high expressions of NER and BER genes suggest that more oxidative DNA lesions are being generated, which lead to genome instability and poor prognosis (Melis et al., 2013). Therefore, the imbalance of DNA damage and repair can increase the genome instability and promote tumor cell proliferation which might contribute to worse survival.

The use of adjuvant therapy in early-stage (IA–IIB) lung adenocarcinoma is controversial in NCCN guidelines and mainly depends on the physician’s experience (Zheng and Bueno, 2015). Although several studies have constructed various gene expression signatures to stratify LUAD patients, none of them have provided sufficient evidence about whether the high-risk patients could benefit from adjuvant therapy (Chen et al., 2007; Shedden et al., 2008; Sun et al., 2008). In our study, we classified LUAD patients into DRG1 and DRG2 subtypes and explored the interaction between these subtypes and adjuvant therapy in the GSE31210 dataset, which was a relatively rigorous clinical trial with clear inclusion and exclusion criteria. The patients in GSE31210 received no neoadjuvant therapies before surgery, whose stages were pathologically defined. Based on the GSE31210 cohort, we found that in the DRG2 subtype, the prognosis of patients who received adjuvant therapy had prolonged survival than those who did not, whereas in the DRG1 subtype, the patients without adjuvant therapy had better prognosis. Several previous studies also revealed that the low activity of TLS including low expression of *REV3L* enhanced the chemosensitivity of cancer (Wang et al., 2015; Yang et al., 2015; Agulló-Ortuño et al., 2020), which supports our findings that patients in the DRG2 subtype may benefit from adjuvant chemotherapy. In summary, the different clinical benefits of adjuvant therapy in various subtypes suggest that DRG subtypes have the potential to guide the selection of adjuvant therapy for early-stage LUAD patients.

In the present study, we identified novel DDR-gene expression subtypes and explored the association with prognosis and adjuvant therapy. However, there are still some limitations in our study. The current study was a retrospective analysis with a limited sample size in a public database. In addition, the mRNA expression in our study was based on RNA-seq or microarray whose results were less stable than those of RT-PCR or IHC, so the evidence would be more solid if it was validated by RT-PCR or IHC, as well as more cost-effective in clinical application scenarios (Pisapia et al., 2022). However, our findings have been validated in six independent cohorts to reduce false-positive results, and they were further validated in the adjuvant therapy subgroups to confirm the role in guiding therapy selection. In the future, prospective studies with large sample sizes are required to confirm the clinical utility of the 16 DDR-gene expression subtypes on the platform of RT-PCR or IHC.

In summary, we explored the association between DDR gene expression and prognosis in patients with stage I or II LUAD. Sixteen DDR gene–related subtypes were constructed to predict prognosis and guide the use of adjuvant therapy. More research studies are warranted to further confirm the clinical utility of the 16 DDR-gene classifiers.

## Data Availability

The original contributions presented in the study are included in the article/Supplementary Material; further inquiries can be directed to the corresponding authors.
