# Supplementary material for: DNA Damage Response Gene-Based Subtypes Associated With Clinical Outcomes in Early-Stage Lung Adenocarcinoma
Source: Front Mol Biosci. 2022 Jun 22;9:901829. doi: 10.3389/fmolb.2022.901829 (PMC9257065; doi:10.3389/fmolb.2022.901829)
Supplement: Supplementary file 1 [file Table1.DOCX]

Supplementary Material

## Supplementary Figures


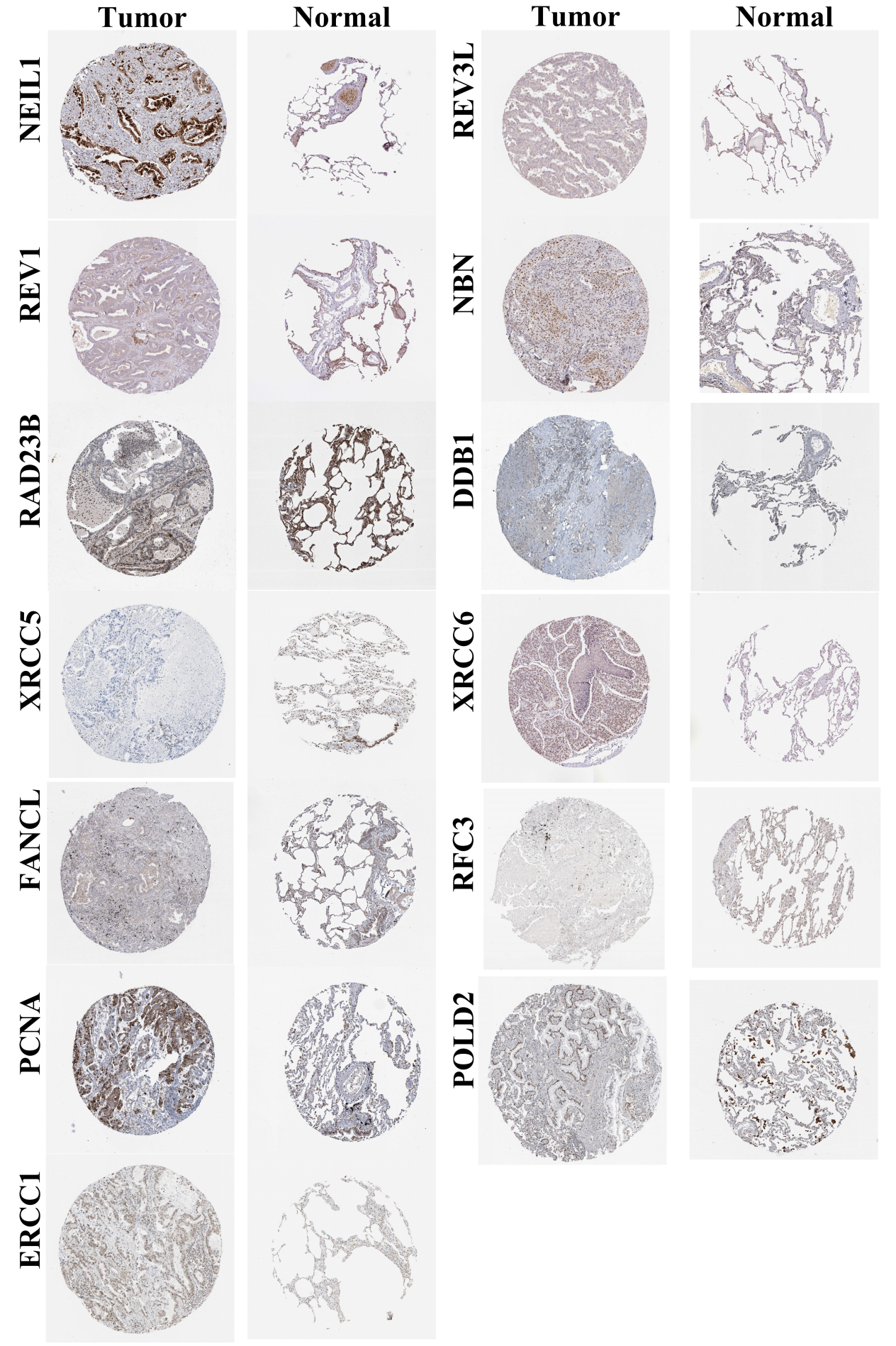


**Supplementary Figure S1. The protein expression of the 13 candidate genes.**


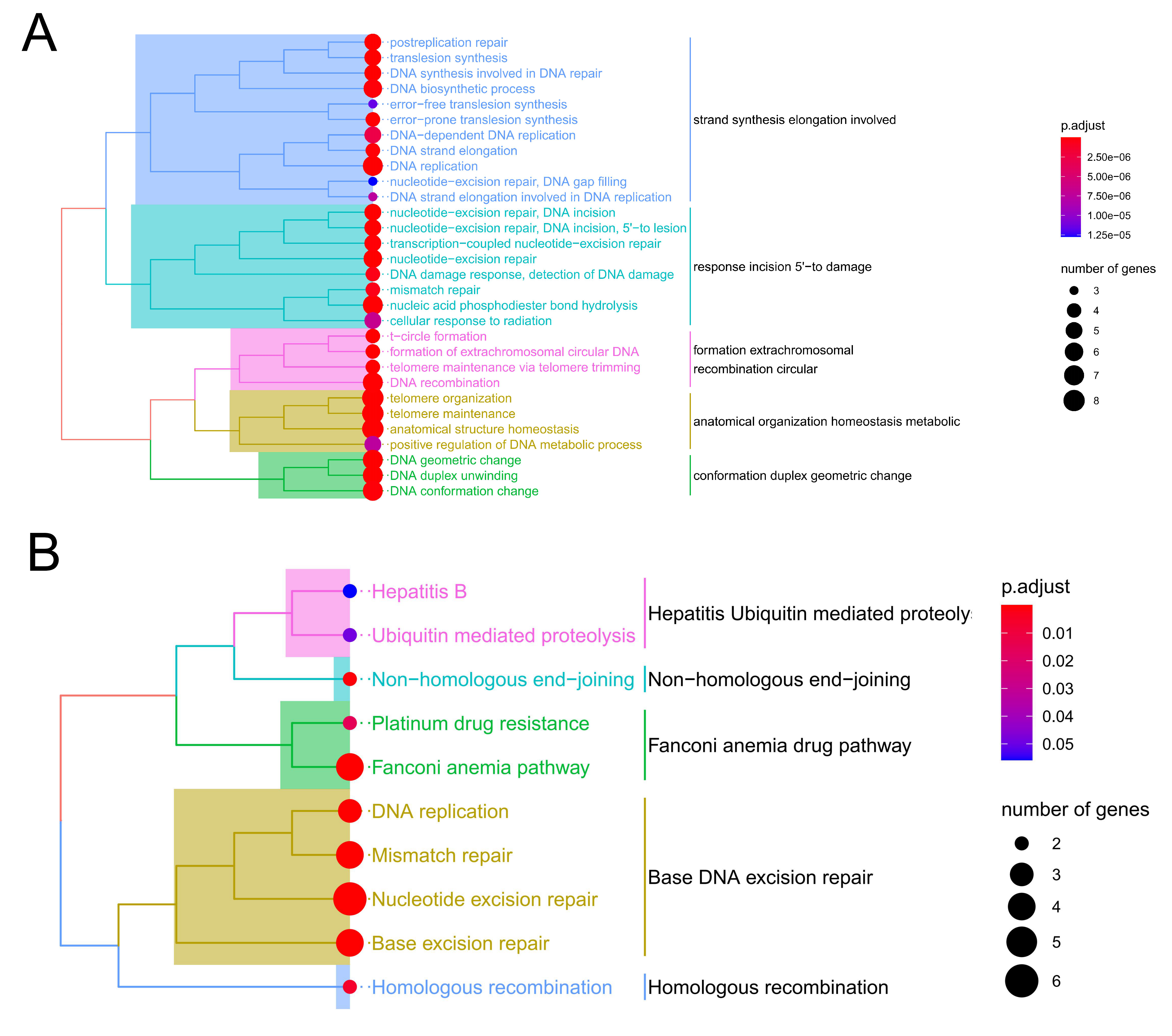


**Supplementary Figure S2.** The Gene enrichment analysis of 16 candidate genes. (A) GO analysis (B) KEGG analysis.


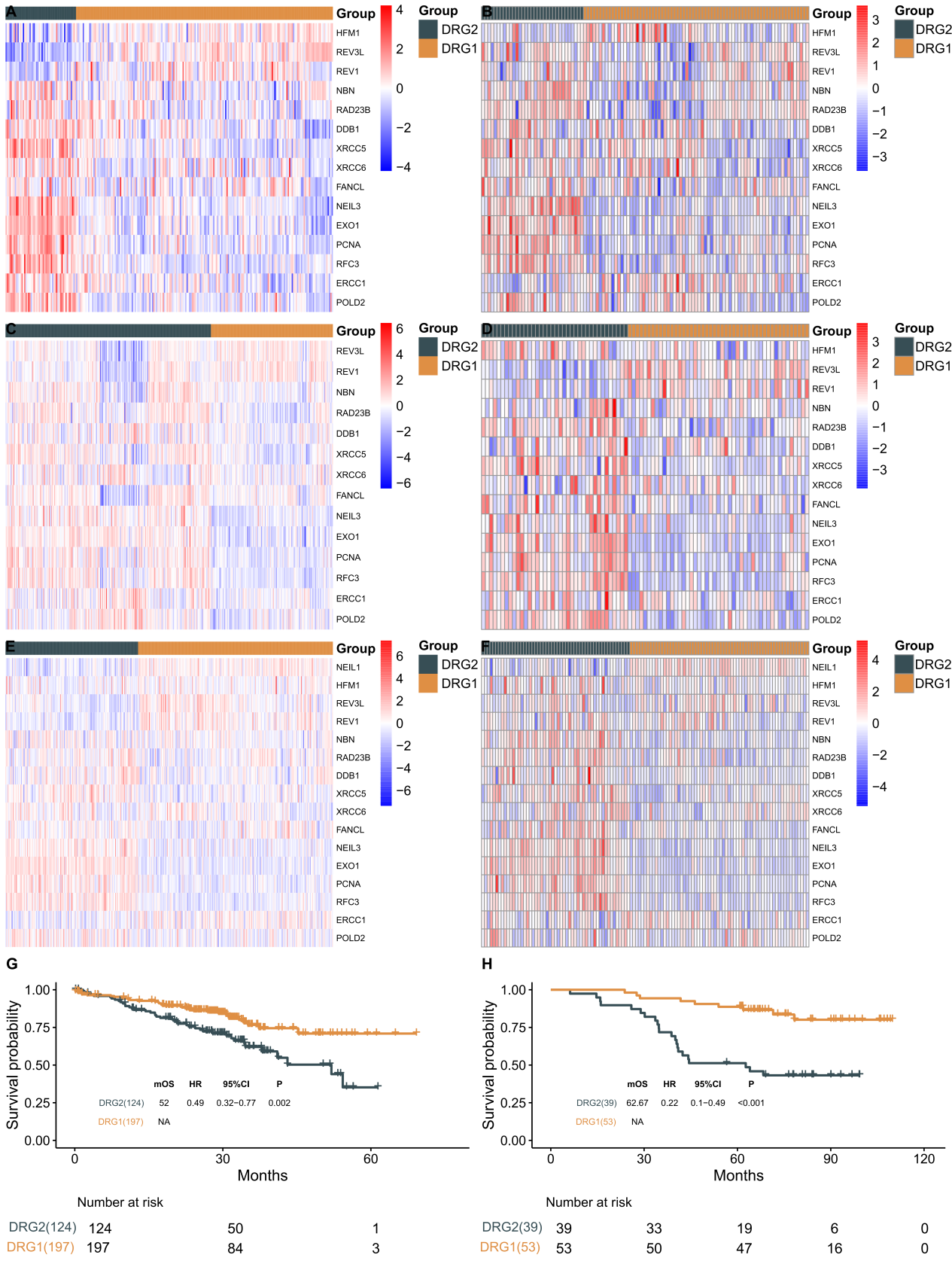


**Supplementary Figure S3.** **The performance of the 16 genes in the validation cohorts**. A-F) The expression pattern of 16 genes in the 6 GEO dataset. G-H) Kaplan-Meier curves showing overall survival between DRG1 (yellow) and DRG2 (grey) in GSE72094 and GSE13213.


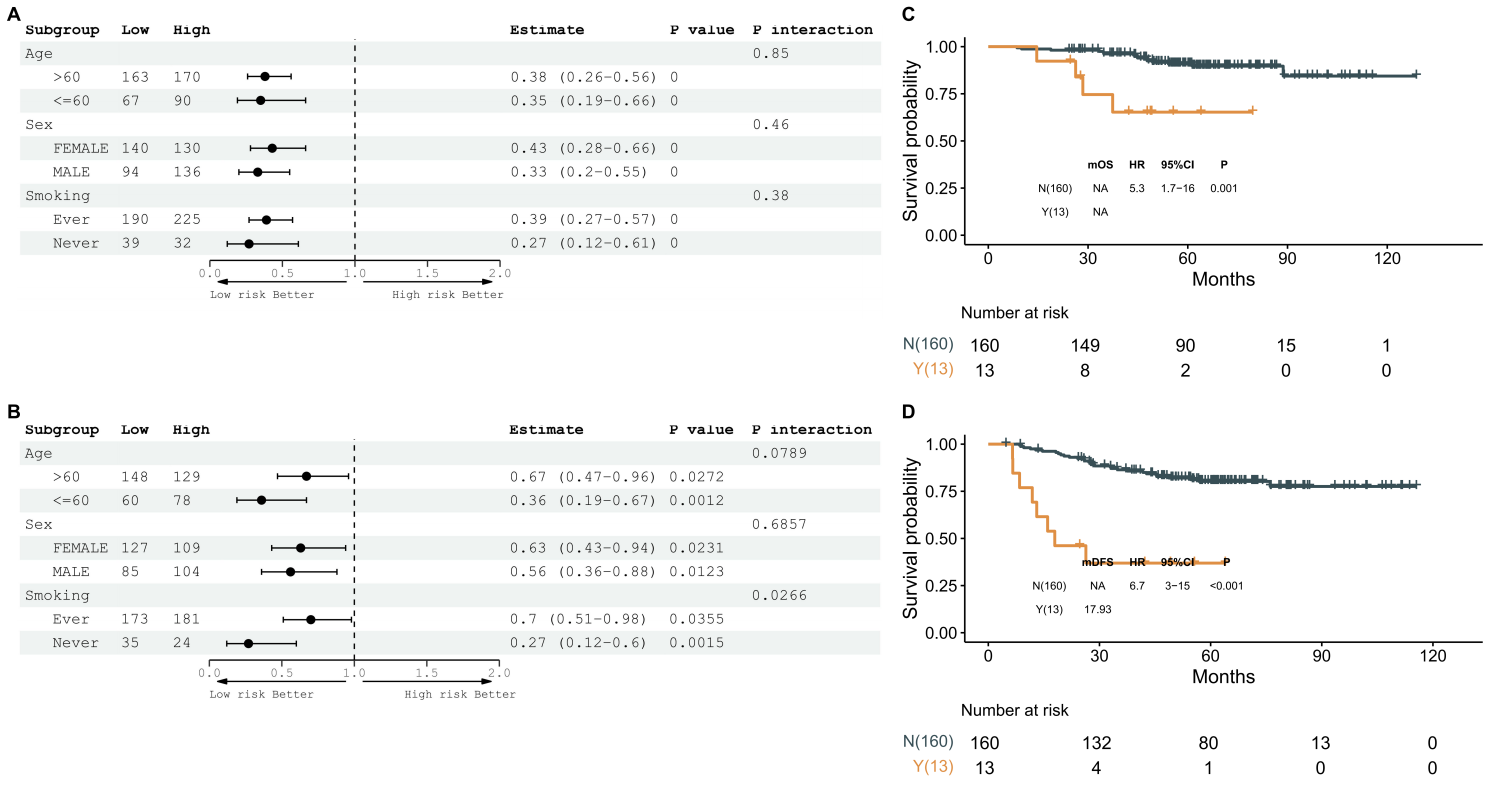


**Supplementary Figure S4. The expression pattern of 16 genes is a prognostic biomarker.** A) Subgroup analyses of overall survival to estimate clinical prognostic value between DRG1 and DRG2 as independent clinical factors in TCGA-LUAD cohort. B) Subgroup analyses of disease-free survival to estimate clinical prognostic value between DRG1 and DRG2 in independent clinical factors in TCGA-LUAD cohort. C) Kaplan-Meier curves of overall survival between patients treated with or without adjuvant therapy in DRG1 of GSE31210 dataset. D) Kaplan-Meier curves of disease-free survival between patients treated with or without adjuvant therapy in DRG1 of GSE31210 dataset.


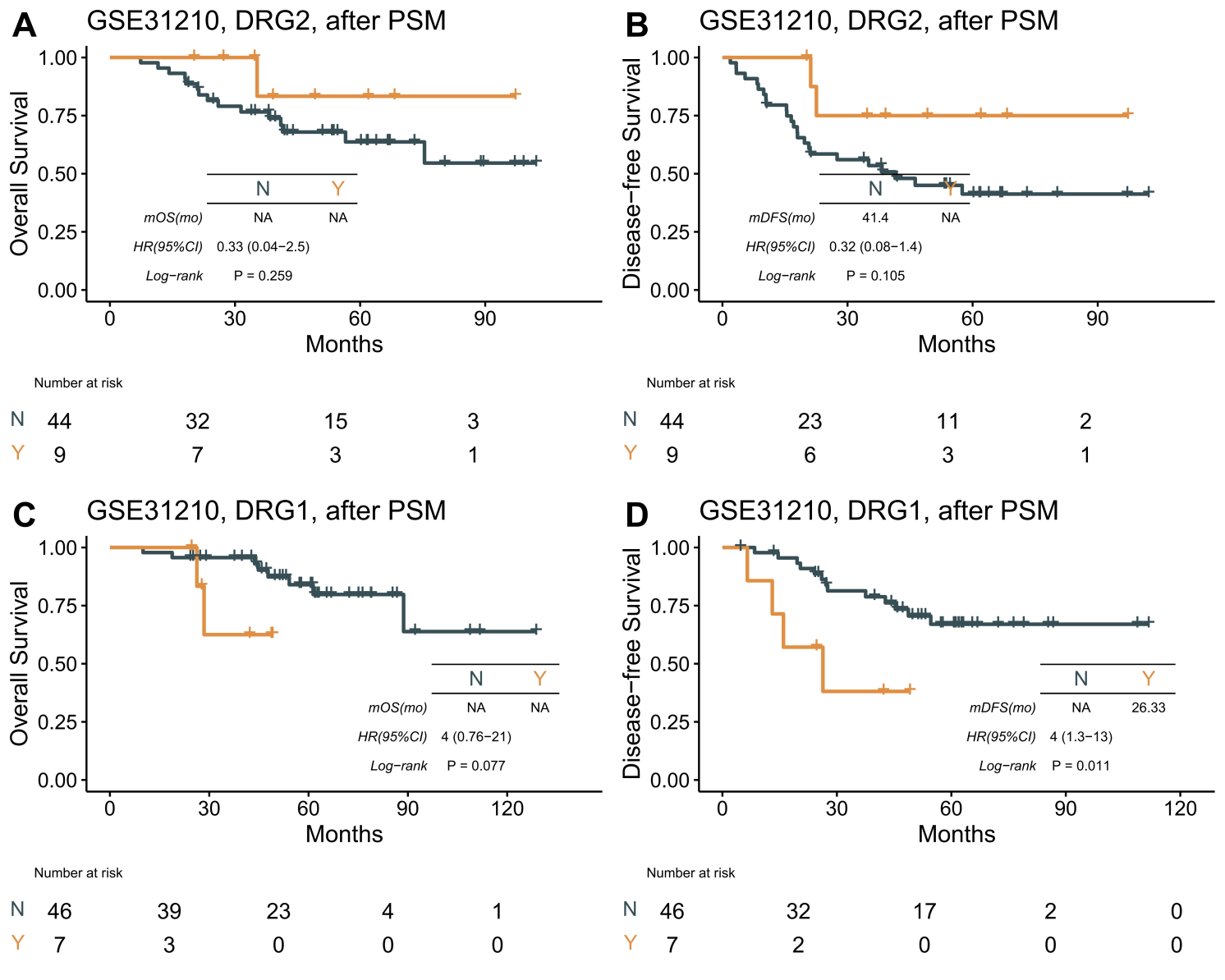


**Supplementary Figure S5. Survival analysis of patients with or without adjuvant therapy in the two DRG subtypes after matched by the propensity score.** A) Kaplan-Meier curves of overall survival between patients treated with or without adjuvant therapy in the DRG2 subtype. B) Kaplan-Meier curves of disease-free survival between patients treated with or without adjuvant therapy in the DRG2 subtype. C) Kaplan-Meier curves of overall survival between patients treated with or without adjuvant therapy in the DRG1 subtype. D) Kaplan-Meier curves of disease-free survival between patients treated with or without adjuvant therapy in the DRG1 subtype.

## Supplementary Tables

**Supplementary Table S1.** Gene list of DNA damage response pathway.

| **Pathway** | **Genes** |
| --- | --- |
| BER (n = 37) | APEX1, APLF, APTX, CCNO, FEN1, HMGB1, LIG1, LIG3, MBD4, MPG, MUTYH, NEIL1, NEIL2, NEIL3, NTHL1, OGG1, PARP1, PARP2, PARP3, PARP4, PCNA, PNKP, POLB, POLD1, POLD2, POLD3, POLD4, POLE, POLE2, POLE3, POLE4, POLL, SMUG1, TDG, TDP1, UNG, XRCC1 |
| CPF (n = 22) | AEN, ATM, ATR, ATRIP, CHEK1, CHEK2, HUS1, HUS1B, PER1, PER2, PER3, RAD1, RAD17, RAD9A, RAD9B, RFC2, RFC3, RFC4, RFC5, TIMELESS, TIPIN, TP53 |
| FA (n = 30) | CENPS, BLM, BRCA1, BRCA2, BRIP1, FAAP100, FAAP24, FAN1, FANCA, FANCC, FANCD2, FANCE, FANCF, FANCG, FANCI, FANCL, FANCM, HES1, PALB2, RAD51, RAD51C, RMI1, RMI2, CENPX, TELO2, TOP3A, TOP3B, UBE2T, USP1, WDR48 |
| HRR (n = 44) | BLM, BRCA1, BRCA2, DMC1, EME1, EME2, GEN1, HFM1, MRE11, MUS81, NBN, PPP4C, PPP4R1, PPP4R2, PPP4R4, RAD50, RAD51, RAD51B, RAD51C, RAD51D, RAD52, RAD54B, RAD54L, RAD54L2, RDM1, RECQL, RECQL4, RECQL5, RMI1, RMI2, RPA1, RPA2, RPA3, SEM1, SLX1A, SLX4, PPP4R3A, PPP4R3B, SPO11, TOP3A, TOP3B, WRN, XRCC2, XRCC3 |
| MMR (n = 25) | EXO1, HMGB1, LIG1, MLH1, MLH3, MSH2, MSH3, MSH4, MSH5, MSH6, PCNA, PMS1, PMS2, POLD1, POLD2, POLD3, POLD4, RFC1, RFC2, RFC3, RFC4, RFC5, RPA1, RPA2, RPA3 |
| NER (n = 47) | BIVM-ERCC5, CCNH, CDK7, CUL3, CUL4A, CUL5, DDB1, DDB2, ERCC1, ERCC2, ERCC3, ERCC4, ERCC6, ERCC8, GTF2H1, GTF2H3, GTF2H4, GTF2H5, LIG1, MMS19, MNAT1, POLR2A, POLR2B, POLR2C, POLR2D, POLR2E, POLR2F, POLR2G, POLR2H, POLR2I, POLR2J, POLR2J2, POLR2K, POLR2L, RAD23A, RAD23B, RBX1, RPA1, RPA2, RPA3, ELOC, ELOB, ELOA, ELOA2, ELOA3B, XPA, XPC |
| NHEJ (n = 15) | APLF, APTX, DCLRE1C, DNTT, LIG4, MRE11A, NHEJ1, POLB, POLL, POLM, PRKDC, RAD50, XRCC4, XRCC5, XRCC6 |
| TLS (n = 13) | HLTF, POLH, POLI, POLK, POLN, RAD18, REV1, REV3L, TMEM189, UBE2B, UBE2N, UBE2V1, UBE2V2 |

**Supplementary Table S2.** Characteristics of TCGA-LUAD cohort and 6 GEO cohorts.

| **Variables** | **GSE13213, N = 184** | **GSE30219, N = 84** | **GSE31210, N = 226** | **GSE37745, N = 89** | **GSE68465, N = 371** | **GSE72094, N = 334** | **TCGA, N = 500** |
| --- | --- | --- | --- | --- | --- | --- | --- |
|  |  |  |  |  |  |  |  |
| **Age (Median (IQR)** | 61 (56, 68) | 60 (55, 69) | 61 (55, 65) | 64 (54, 70) | 65 (58, 72) | 70 (64, 77) | 66 (59, 72) |
| **Sex, n (%)** |  |  |  |  |  |  |  |
| **FEMALE** | 84 (46%) | 19 (23%) | 121 (54%) | 50 (56%) | 188 (51%) | 179 (54%) | 270 (54%) |
| **MALE** | 100 (54%) | 65 (77%) | 105 (46%) | 39 (44%) | 183 (49%) | 155 (46%) | 230 (46%) |
| **Smoking, n (%)** |  |  |  |  |  |  |  |
|  | 0 (0%) | 84 (100%) | 0 (0%) | 89 (100%) | 74 (20%) | 51 (15%) | 14 (2.8%) |
| Ever | 94 (51%) | 0 (0%) | 111 (49%) | 0 (0%) | 256 (69%) | 256 (77%) | 415 (83%) |
| Never | 90 (49%) | 0 (0%) | 115 (51%) | 0 (0%) | 41 (11%) | 27 (8.1%) | 71 (14%) |
| **Tumor Stage,n (%)** |  |  |  |  |  |  |  |
|  | 0 (0%) | 0 (0%) | 0 (0%) | 0 (0%) | 0 (0%) | 0 (0%) | 8 (1.6%) |
| I | 158 (86%) | 71 (85%) | 168 (74%) | 70 (79%) | 276 (74%) | 265 (79%) | 268 (54%) |
| II | 26 (14%) | 13 (15%) | 58 (26%) | 19 (21%) | 95 (26%) | 69 (21%) | 119 (24%) |
| III | 0 (0%) | 0 (0%) | 0 (0%) | 0 (0%) | 0 (0%) | 0 (0%) | 80 (16%) |
| IV | 0 (0%) | 0 (0%) | 0 (0%) | 0 (0%) | 0 (0%) | 0 (0%) | 25 (5.0%) |

**Supplementary Table S3.** The basic description of samples with IHC images.

| **Patient_ID** | **Age** | **Sex** | **Gene** | **Sample Type** | **Antibody** |
| --- | --- | --- | --- | --- | --- |
| PID3052 | 51 | Female | DDB1 | Normal | CAB032821 |
| PID2208 | 67 | Female | DDB1 | LUAD | CAB032821 |
| NA | NA | NA | EXO1 | Normal | NA |
| NA | NA | NA | EXO1 | LUAD | NA |
| PID2268 | 49 | Female | ERCC1 | Normal | CAB004390 |
| PID847 | 64 | Male | ERCC1 | LUAD | CAB004390 |
| PID3076 | 20 | Male | FANCL | Normal | HPA036685 |
| PID687 | 65 | Male | FANCL | LUAD | HPA036685 |
| NA | NA | NA | HFM1 | Normal | NA |
| NA | NA | NA | HFM1 | LUAD | NA |
| PID2268 | 49 | Female | NBN | Normal | HPA001429 |
| PID303 | 68 | Male | NBN | LUAD | HPA001429 |
| PID1678 | 57 | Female | NEIL1 | Normal | HPA054084 |
| PID448 | 76 | Female | NEIL1 | LUAD | HPA054084 |
| NA | NA | NA | NEIL3 | Normal | NA |
| NA | NA | NA | NEIL3 | LUAD | NA |
| PID2268 | 49 | Female | PCNA | Normal | HPA030522 |
| PID1847 | 64 | Male | PCNA | LUAD | HPA030522 |
| PID2268 | 49 | Female | POLD2 | Normal | HPA026745 |
| PID3391 | 70 | Feamle | POLD2 | LUAD | HPA026745 |
| PID3076 | 20 | Male | RAD23B | Normal | CAB033868 |
| PID2041 | 51 | Female | RAD23B | LUAD | CAB033868 |
| PID1678 | 57 | Female | REV1 | Normal | HPA051036 |
| PID4365 | 71 | Male | REV1 | LUAD | HPA051036 |
| PID1678 | 57 | Female | REV3L | Normal | HPA064853 |
| PID1847 | 64 | Male | REV3L | LUAD | HPA064853 |
| PID3076 | 20 | Male | RFC3 | Normal | HPA030149 |
| PID1687 | 65 | Male | RFC3 | LUAD | HPA030149 |
| PID2101 | 21 | Male | XRCC5 | Normal | CAB004468 |
| PID2041 | 51 | Female | XRCC5 | LUAD | CAB004468 |
| PID2208 | 67 | Female | XRCC6 | Normal | HPA047549 |
| PID3003 | 49 | Male | XRCC6 | LUAD | HPA047549 |
